# Morphometric study of percutaneous A1 pulley of thumb release

**DOI:** 10.1038/s41598-022-24759-5

**Published:** 2022-12-05

**Authors:** Wei-xing Zhong, Zu-jiang Chen, Wei-jie Peng, Rui-bin Gu, Jun-hua Li, Yi-kai Li

**Affiliations:** School of Traditional Chinese Medicine, Southern Medical University, Guangzhou, Guangdong China

**Keywords:** Anatomy, Diseases, Pathogenesis

## Abstract

Through anatomy, microscope, histopathology, and simulating needle knife operation on specimens, to accumulate the relevant parameters of the A1 pulley of thumb, and to provide an anatomical evidence for the needle knife therapy of stenosing flexor tenosynovitis. A total of 20 fingers were selected from 20 intact adult upper limb specimens, a small amount of emerald green waterproof dye was injected from the needle insertion point, dissected layer by layer, and the A1 pulley and neurovascular bundle were observed. Observe the loosening of the thumb A1 pulley after 5 and 10 times of simulated needle knife cutting on the specimen; observe the relationship between the needle knife entry point and the A1 pulley under the thumb extension and abduction, and the thumb extension neutral position respectively; further observe the histological characteristics, and the relationship between needle entry point and A1 pulley by microscope. ① In general observation, the A1 pulleys of each finger were transverse fibers perpendicular to the flexor tendon, tough in texture, connected with synovial fibers at the proximal end. It is difficult to distinguish, and connected with oblique fibers at the distal end. ② The release rate of the thumb A1 pulley after 5 and 10 times of simulated needle knife cutting on the specimen were (40.46 ± 2.22)% and (63.52 ± 4.49)%, respectively. ③ In the neutral position of the thumb straightening, the needle entry point is 3.06 ± 0.14 mm from the proximal side of the proximal edge of the A1 pulley, which overlaps with the needle entry point where the thumb is straight and abducted. ④ Observed under a microscope, the A1 pulley is a dense transverse fiber with a pale yellow dense connective tissue, both ends are continuous with the synovial fibers. It is thin and translucent, and loose connective tissue. The A1 pulley is a dense transverse fiber with a pale yellow dense connective tissue. The anatomical key points of the needle knife therapy lie in the extended and abducted position of the thumb. Currently, it is believed that cutting the proximal edge of the A1 pulley is sufficient, and there is no need to cut the entire A1 pulley.

## Introduction

Trigger finger (TF), also called stenosing flexor tenosynovitis, can be described as a difference in diameters of the flexor tendon and the A1 pulley due to thickening and narrowing of the tendon sheath, located at the metacarpal head^1^. TF is one of the most common diseases of the hand occurring in 2–3% of the population^2^. The most common finger is the thumb^3^.

The treatment for TF includes conservative treatment and operative treatment. The former includes the use of nonsteroidal anti-inflammatory medications for pain control, corticosteroid injections, and splints, and also includes Traditional Chinese Medicine such as acupuncture, massage, plaster therapy, while the latter includes percutaneous release and open release^4^. Surgical treatment is highly successful and widely regarded as the ultimate treatment for TF. However, as shown in literature reviews, percutaneous blind A1 pulley release is an alternative to the open release, but its risk–benefit relationship is under debate^5^.

A needle knife is a traditional tool of Chinese medicine that has been used widely by the rehabilitation doctors of China for thousands of years ago. TF is the preferred alternative for the needle knife^6^.

This paper uses anatomy, microscope, histopathology, and other techniques to observe the A1 pulley-related parameters on 20 adult upper limb specimens and simulate needle knife operation on the specimens. To provide an anatomical basis for acupuncture treatment of flexor tendon stenosing tenosynovitis, to improve the accuracy, effectiveness, and safety of treatment.

## Materials and methods

The present study was performed in fresh frozen adult human cadavers that have been donated to the Department of Anatomy, Southern Medical University (SMU), Guangzhou through the Institutional body donation program following the ethical guidelines. Informed consent taken from the donor or next to kin. The protocol for this research project was approved by the Institute Ethics committee, SMU, and all methods were performed in accordance with the relevant guidelines and regulations. The study design involved the dissection of 20 preserved cadaver hands (Table 1). The study period was from April 2021 to December 2021. All specimens had intact hands, no deformity, no damage, and no history of surgery.

**Table 1:** General characteristics of digits.

No. digits	20
Right/left	10/10
Thumb	20

### Main reagents, instruments and tools

**Figure 1:**
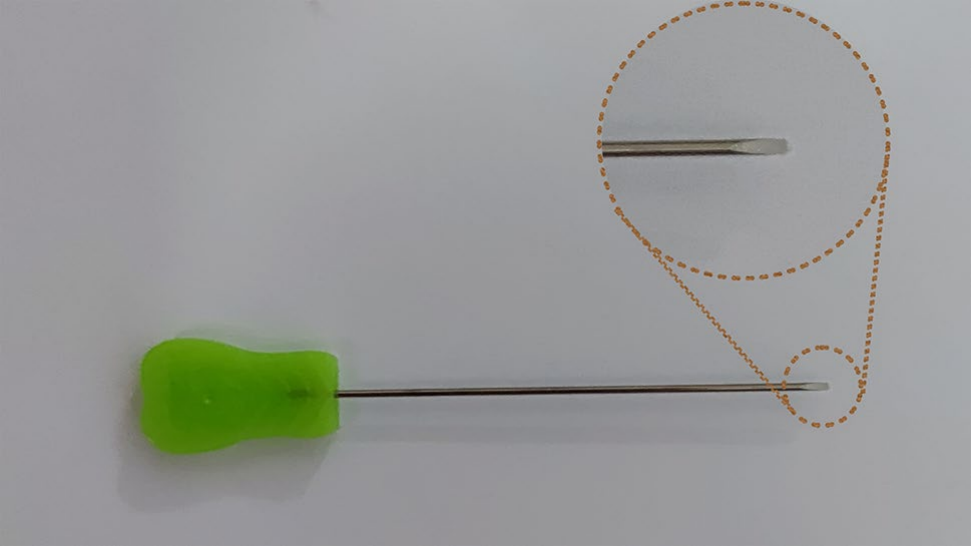
The Hanzhang needle knife. The needle knife consists of three parts: tip, body and handle, with a 1.2 mm blade on the tip, and a 50 mm length of the body.

Hematoxylin–eosin (HE) staining kit; pathological slicer (Shanghai Leica Instruments Co., Ltd.), Olympus BX51 upright microscope (Olympus, Japan); electronic vernier calipers (Accuracy 0.01 mm), ophthalmic scissors, ophthalmic forceps, scalpel, hemostatic forceps, skull gauge, marker, digital image acquisition (D610 camera, Nikon Corporation), image processing (photoshop 2020/Adobe Illustrator 2020, Adobe Corporation ), needle knife ( Hanzhang Medical Instrument Co., Ltd., 1.2 × 50 mm) (Fig. 1).

#### Step 1

Simulate the operation of the needle knife on the thumb to treat the trigger finger, take the point P of the thumb as the needle insertion point (the metacarpophalangeal joint and the transverse pattern of the metacarpophalangeal finger overlap), insert the needle vertically along the M line(the midline of the palm face of thumb), cut 5 times or 10 times to the distal end. Then thumb was dissected in turn, the skin was incised along M line, the soft tissues were separated layer by layer from superficial to deep, and soft tissues such as nerves, blood vessels, flexor tendons, and tendon sheaths were exposed, photographed and recorded.

#### Step 2

**Figure 2:**
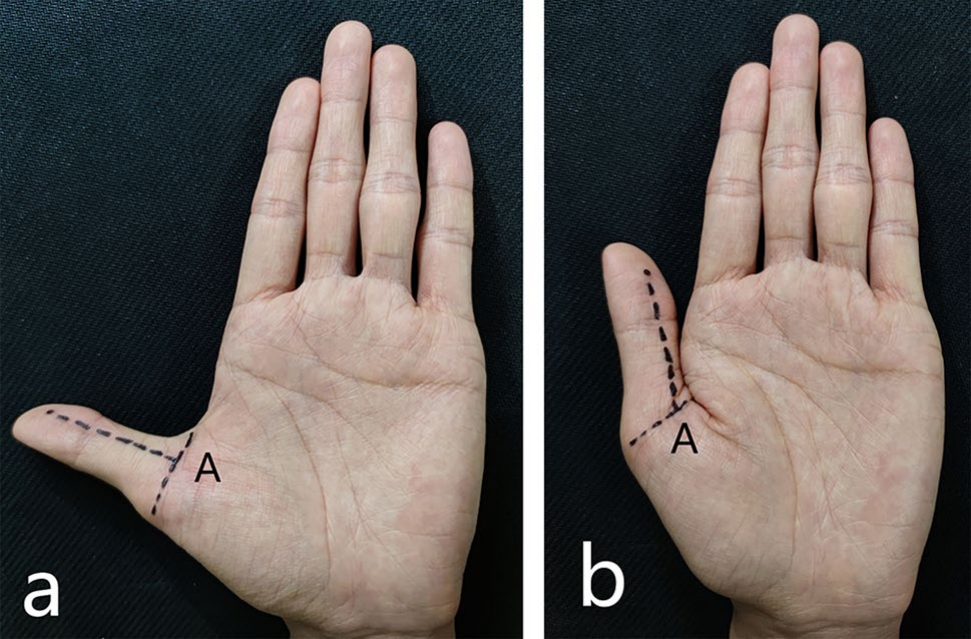
Two positions of the thumb: (**a**) extension and abduction; (**b**) neutral extension.

IN two positions of the thumb (extension and abduction, neutral extension) (Fig. 2), emerald green dye and yellow dye were used to locate the needle entry point, respectively. Then observed the difference between the needle entry point and the proximal edge of the A1 pulley.

#### Step 3

Finally, the tendon sheaths of each finger were removed. And observed the histological characteristics of the A1 pulley by HE staining.

### Description of the measurement parameters

The A1 pulley, flexor tendon, and surrounding soft tissue structures were observed, and the following anatomical structures were measured using an electronic vernier caliper: the distance (D) of the midpoint of the proximal border of the A1 pulley (B) and the needle entry point (A), the length of the A1 pulley of thumb, the length of the thumb A1 pulley to be cut. The unit of distance is millimeters (mm).

### Data analysis

Depending on the type of variable, mean values, SEM values, ranges, absolute frequencies, and percentages were recorded. Analysis of variance, the Chi-square test, and the paired *t* test were used to analyze differences. A 0.05 level of statistical significance was considered significant.

### Ethics declarations

Informed consent was obtained from all subjects and/or their legal guardians.

### Consent to participate

All authors agree to participate.

## Results

### Observation of HE staining

**Figure 3:**
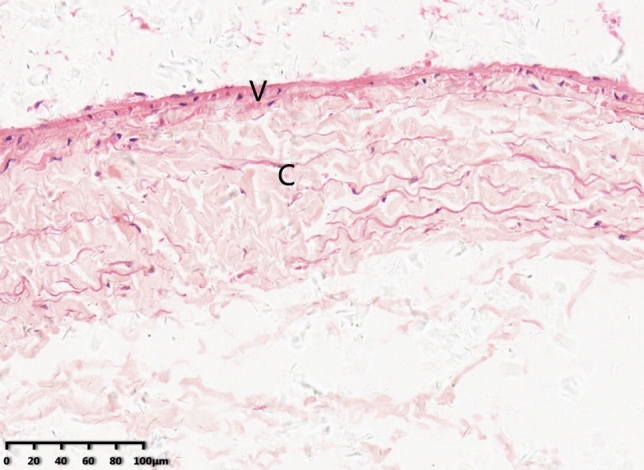
The A1 pulley, thumb, transverse section (hematoxylin–eosin stain). The pulley is composed of two layers: a vascular, outer, frictionless layer *(V)* and a collagenous layer *(C)*. (Original magnification×200).

Observed under the microscope, the A1 pulley of thumb is composed of two layers: a vascular, outer, frictionless layer and a collagenous layer. This is consistent with what we see under the stereo microscope, the A1 pulley is a dense transverse fiber with a pale yellow dense connective tissue, and both ends are continuous with the synovial fibers (Fig. 3).

### The rate of releasing thumb A1 pulley

**Figure 4:**
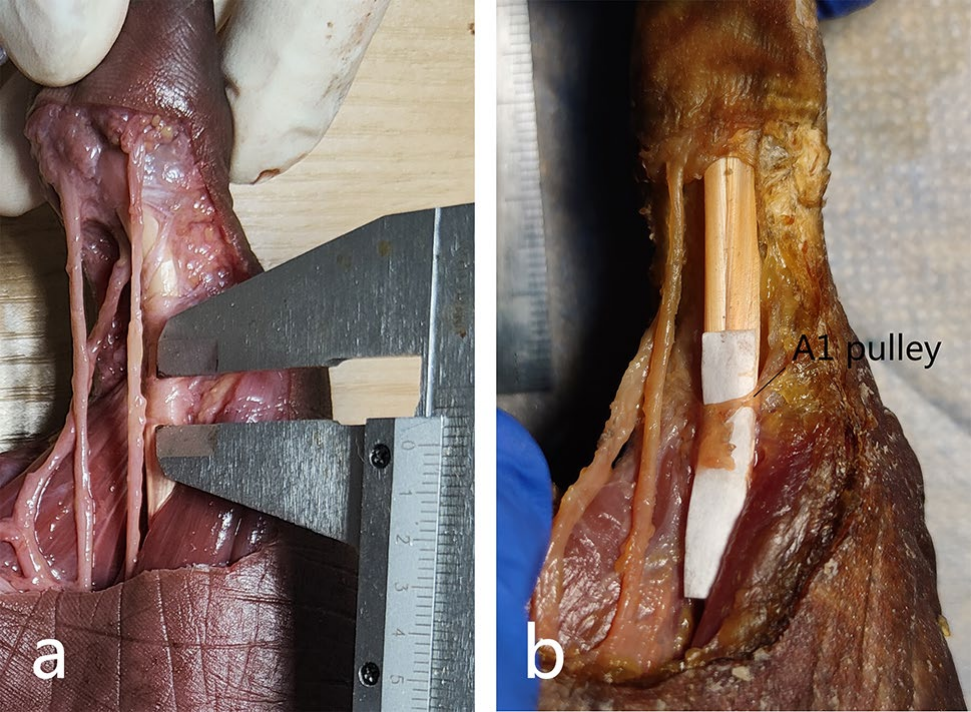
(**a**) The A1 pulley of thumb; (**b**) the A1 pulley after cutting 10 times.

The needle knife simulates cutting the thumb A1 pulley, cutting 5 times and 10 times respectively. The release rate of the two is compared as shown in Table 2. After cutting 5 times *(40.46* ± *2.22)%*, cutting 10 times *(63.52* ± *4.49)%*, both statistically significant differences *(P* < *0.001)* (Fig. 4)*.*In addition, it was generally observed that the cut marks of the A1 pulley did not maintain a straight line, and the flexor pollicis longus tendon had longitudinal lacerations.

**Table 2:** The Rate of releaseing Thumb A1 pulley.

	Length of thumb A1 pulley (mm)	Length of cutting (mm)	Percent of releaseing (%)
5 times	6.20 ± 0.26	2.51 ± 0.19	40.46 ± 2.22
10 times	6.11 ± 0.37	3.88 ± 0.33	63.52 ± 4.49
F	0.216	65.352	105.827
P	0.655	0.001	0.001

### The distance between A and B in different positions of thumb

The distance between A and B of the thumb is shown in Table 3. In the neutral position with the thumb extended, the distance is *3.06* ± *0.14 mm*; the thumb is extended and abducted position, A overlaps B.

**Table 3:** The distance between A and B in different positions of thumb *(*$\overline{x }$±*s, mm)*.

Position	D
Extended and neutral position	3.06 ± 0.14
Extended and abducted position	0

## Discussion

### Histological characteristics and adjacent relationship of the A1 pulley

Literature has shown that the A1 pulley is composed of two layers: an outer (convex, vascular layer) and an inner (concave, friction, avascular, gliding layer)^7^. Our study also supports this conclusion. More importantly, the inner gliding layer of the A1 pulley has many fibrocartilaginous characteristics. The fabric of the friction layer contains chondrocytes, not synovial cells. This is similar to the synovial joint in the human body, providing the conditions for the glide of the tendon.

Previous studies have shown that increased compressive forces on connective tissues result in fibrocartilaginous metaplasia^8^. Electron microscopy results suggest that the friction forces provoke areas of cartilaginous transformation, or metaplasia, in the deeper part of the gliding layer^9^. This suggests to us that the pathogenesis of TF is probably due to the fibrocartilaginous metaplasia in the A1 pulley, which may produce the difference in diameters of the tendon sheath and flexor tendon, especially stenosis proximal to the A1 pulley. Therefore, in clinical practice, the proximal end of the A1 pulley is often cut, but not all, to cure TF. Literature suggests that operative success or failure is determined mainly by the complete or incomplete release of the proximal portion of the pulley and that the distal portion of the pulley does not necessarily correlate with clinical outcome^10^.

### Length of needle knife release

Kinds of literature have shown us that TF may occur due to a mismatch between A1 pulley and flexor tendon which impairs the smooth gliding of the tendon inside it. This may occur due to flexor tendinosis with a nodule or thickening of A1 pulley or tenosynovitis^11^. The A1 pulley is the most affected pulley as it is the site of maximal tendon excursion^12^. In addition, high pressures occur at the proximal edge of the A1 pulley on maximal flexion and during tight grip. On the other hand, the literature suggests that operative success or failure is determined mainly by the complete or incomplete release of the proximal portion of the pulley and that the distal portion of the pulley does not necessarily correlate with clinical outcome^13^. In fact, it may not be necessary to completely divide the A1 pulley to abolish the triggering^14^. Therefore, it is only necessary to release the proximal edge of the A1 pulley.

Excessive length of release results in the incision of the proximal border of the A2 pulley (22%, mean 2.3 mm)^15^, which may be inconsequential and insufficient to cause any functional deficits. However, complications such as bowstring pain cannot be ruled out. The knowledge of gap distances can play a significant role in percutaneous or minimally invasive surgeries of the pulleys to avoid excessive release and injury to the adjacent pulley leading to bowstringing or under-release causing recurrence of TF^15^. Therefore, we need to further study the distance between the pulleys.

Excessive release may lead to complications such as bowstring pain. Too narrow a release range may increase the recurrence rate. How to standardize the release length is the key issue in the operation. Currently, it is tentatively believed that cutting the proximal edge of the A1 pulley is sufficient, and there is no need to cut the entire A1 pulley. It does not need to extend to the A2 pulley, and the specific number of cuts and the length of the cut are determined by whether the patient’s TF is released during the operation.

### Patient position

Our study shows that with the thumb in the extended abduction position, the needle knife is safer and more convenient to cut the A1 pulley, and it is easier to cut the tendon sheath. From a safety point of view, the palm is up and the neutral position is straight, the metacarpophalangeal joint is hyperextended, which is convenient for the needle knife to enter, and can ensure the maximum distance from the A2 pulley. The A1 pulley is stretched and is in a more superficial position, and the neurovascular is safer away from tendon sheaths^13^. It is worth noting that there is a literature reminder that the radial digital nerve of the thumb is close to the flexor tendon at the level of the A1 pulley^11^, which is likely because the thumb is in the neutral position of extension and not in the extension and abduction position. Our research shows that the flexor pollicis longus tendon has a large distance (5.45–11.15 mm) from the neurovascular bundles on both sides. As long as the correct needle insertion site is ensured, it is generally difficult to injure.

From an effectiveness point of view, the thumb are in a straight and abduction position, the A1 pulley is stretched, tauter, and maintains a stable extension and a certain tension, which is beneficial to the needle knife for cutting. Because the skin and subcutaneous tissue are tight and not easy to move, which helps marking the positioning of the body surface and the direction of cutting, and the feeling under the needle is obvious after the needle is inserted. The most important thing is that the A1 pulley is in a more superficial position, which is also good for needle knife cutting.

## Conclusions

The A1 pulley of thumb is composed of two layers: a vascular, outer, frictionless layer and a collagenous layer. The anatomical key points of the needle knife therapy lie in the extended and abducted position of the thumb. Currently, it is believed that cutting the proximal edge of the A1 pulley is sufficient, and there is no need to cut the entire A1 pulley.

## Limitation

There were several limitations in our study, firstly that this study was performed on cadavers and required clinical proof of the efficiency of length of needle knife release and the thumb in the extended abduction position. Secondly, this study didn’t distinguish the sex of the cadaver hands, which may affect the results.

## Data Availability

All data generated or analysed during this study are included in this published article.
